# Isolation, identification, and pathogenicity determination of fungal pathogens causing root rot of *Stipa bungeana* in Inner Mongolia, China

**DOI:** 10.3389/fpls.2026.1936227

**Published:** 2026-09-07

**Authors:** Zhida Liu, Haoran Xie, Luran Wang, Le Wang, Yuanyuan Zhang, Ruifang Jia, Zhengqiang Chen

**Affiliations:** 1 Key Laboratory of Biohazard Monitoring, Green Prevention and Control for Artificial Grassland, Ministry of Agriculture and Rural Affairs, Institute of Grassland Research of Chinese Academy of Agricultural Sciences, Hohhot, China; 2 Inner Mongolia Key Laboratory of Grassland Conservation Ecology, Institute of Grassland Research of the Chinese Academy of Agricultural Sciences, Hohhot, China; 3 College of Horticulture and Plant Protection, Inner Mongolia Agricultural University, Hohhot, China

**Keywords:** *Curvularia spicifera*, first report, Inner Mongolia, pathogenicity, root rot

## Abstract

*Stipa bungeana* Trin. is a dominant grass species in the warm temperate steppes of northern China; however, its root rot diseases have not been systematically investigated. In this study, 100 root samples of *S. bungeana* showing typical root rot symptoms were collected from the Sha’erqin Research Base in Tumd Left Banner, Hohhot City, Inner Mongolia, China. A total of 76 fungal isolates, were recovered and identified based on morphological characteristics and multi-locus sequence analysis of the internal transcribed spacer (ITS) region, translation elongation factor 1-alpha (TEF1-α), and the second largest subunit of RNA polymerase II (RPB2) gene. Eight species were identified, of which *Curvularia spicifera* (25 isolates, 32.89%) and *Microdochium bolleyi* (24 isolates, 31.58%) were the predominant species. Pathogenicity tests conducted on healthy *S. bungeana* seedlings under greenhouse conditions demonstrated that all 8 species induced root rot symptoms, thereby, fulfilling Koch’s postulates. Notably, *C. spicifera* and *M. bolleyi* exhibited significantly greater virulence than the *Fusarium* species and the other recovered fungi, resulting in higher disease incidence and plant mortality. This study represents the first comprehensive report of the fungal pathogens associated with root rot of *S. bungeana* in China and provides the first evidence that *C. spicifera* and *M. bolleyi* are the predominant and most virulent pathogens associated with root rot of *Stipa* species. These findings fill an important critical knowledge gap in grassland plant pathology and provide a scientific foundation for disease management in natural steppe ecosystems.

## 1 Introduction

Grasslands represent one of the Earth’s most extensive terrestrial biomes, covering approximately 40% of the global land surface and playing roles pivotal in global ecological balance (Bardgett et al., 2021; MacDougall et al., 2026), conserving biodiversity (Prangel et al., 2024), and supporting human livelihoods (Hilal et al., 2024). However, grasslands worldwide are facing unprecedented threats from climate change (Bakker et al., 2024), land-use intensification (Wright and Wimberly, 2013), overgrazing (Li and Zhao, 2025), and the increasing prevalence of plant diseases (Seabloom et al., 2010; Ogbuji and Agogbua, 2025). Among these pressures, diseases caused by pathogenic microorganisms can substantially reduce plant biomass (Maron et al., 2011), alter community composition (Bagchi et al., 2014), and compromise the overall health and resilience of grassland ecosystems (Romero et al., 2024). Therefore, the accurate and timely identification of plant pathogens is fundamental to effective disease management and conservation of these vital natural ecosystems.

*Stipa bungeana* Trin., a perennial bunchgrass within the Poaceae family, is a dominant and constructive species in the warm temperate typical and desert steppes of southern Eurasia (Jiang et al., 2025). Endemic to China, this species is predominantly found in the Loess Plateau and its surrounding regions (Wang et al., 2021). The presence of *S. bungeana* is essential for enhancing the regional ecological environment and addressing desertification in the area. Additionally, as a high-quality native forage grass (Wang et al., 2022), *S. bungeana* holds significant value for grazing and the restoration of degraded grasslands in agropastoral ecotones in northwestern China (Zhang et al., 2017). Despite its ecological and economic importance, the natural vegetation of *S. bungeana* has become highly fragmented because of agricultural expansion. Consequently, the protection and restoration of the remaining habitats are of high priority for regional conservation efforts (Shao et al., 2025). Nonetheless, the natural vegetation has undergone severe fragmentation and remains in an unstable successional stage due to agricultural expansion and reclamation. Additionally, similar to many other significant grassland species, these grasses are vulnerable to various diseases (Dang et al., 2025; Gao et al., 2025). Recent studies have begun to shed light on the pathogens affecting these key grasses, such as the identification of *Alternaria alternata* causing leaf spots on *Leymus chinensis* (Wu et al., 2026) and documented rust epidemics caused by Puccinia species on several native grasses in Inner Mongolia (Zhang et al., 2022). These findings highlight the vulnerability of steppe ecosystems to plant pathogens and emphasize the need for comprehensive pathological investigations.

Despite the ecological and economic significance of *Stipa* species, there is a notable paucity of information regarding the diseases affecting these grasses in natural ecosystems. No comprehensive studies have been conducted on root rot diseases affecting *S. bungeana* or other *Stipa* species, either in China or elsewhere. This knowledge gap is particularly concerning, as root rot is a destructive soil-borne disease that damages the root system, impairs water and nutrient absorption, and frequently results in plant wilting and death (Parshuramkar et al., 2026). Root rot can have a particularly severe impact on natural ecosystems, where pathogen inoculum can accumulate in the soil over long periods. For instance, recent studies have identified novel and highly destructive root rot pathogens, such as *Fusarium sibiricum*, infecting *Chamerion angustifolium*, demonstrating the continual emergence of new pathogenic threats (Zhang et al., 2026). Given the current challenges of overgrazing, climate change, and grassland degradation, the incidence and severity of root rot diseases are likely to increase. In contrast, root rot has been extensively studied in alfalfa (*Medicago sativa* L.), the most important forage legume in China. Studies conducted in Inner Mongolia, Ningxia have consistently identified several *Fusarium* species as the primary causal agents of alfalfa root rot including *F. acuminatum*, *F. solani*, *F. equiseti*, *F. oxysporum*, and *F. redolens*. Pathogenicity assays performed under controlled conditions have confirmed that *Fusarium* species consistently induce typical root rot symptoms, with *F. solani* and *F. oxysporum* exhibiting particularly high virulence (Yang et al., 2022). Furthermore, root rot pathogens have been reported from *Astragalus* species and several medicinal plants (Ma et al., 2022).

In early May 2025, field surveys conducted at the Sha’erqin Research Base, Tumd Left Banner, Hohhot, revealed widespread and severe root rot in wild populations of *Stipa bungeana* within this typical agro-pastoral ecotone. Multiple representative patches exhibited obvious disease symptoms: numerous individuals failed to fully regreen in spring and exhibited extensive withering of aboveground tissues, while most clumps displayed uneven tiller chlorosis and partial dieback. Root dissection of symptomatic plants revealed extensive cortical browning, root tissue necrosis, and a marked reduction in root biomass, which was associated with visible degradation of native steppe vegetation cover at the study site. To date, no systematic studies have comprehensively characterized the pathogenic complex responsible for root rot in *S. bungeana* or other *Stipa* species, representing a critical knowledge gap in temperate steppe plant pathology. The research base is located within a typical agropastoral ecotone, a transitional zone between cropland and natural steppe. To identify the pathogenic fungi associated with these symptoms, characterize the fungal community composition associated with root rot, and verify the pathogenicity of the principal isolates through pathogenicity assays, a systematic study was conducted. The study included fungal isolation, morphological and multilocus molecular identification, and pathogenicity testing based on Koch’s postulates. The findings provide the first comprehensive characterization of the causal agents of root rot in *S. bungeana*, addressing an important gap in grassland pathology and providing a scientific basis for the future development of disease management strategies to protect this important steppe ecosystem. Greenhouse pathogenicity assays further demonstrated that the two dominant fungal taxa isolated from diseased roots induced severe disease symptoms and significant seedling mortality,, indicating that root rot may pose a substantial and previously under-recognized threat to the establishment and survival of *S. bungeana.*

## 2 Materials and methods

### 2.1 Sample collection and field symptom observation

In early May 2025, a field survey was conducted to investigate root rot of *Stipa bungeana* Trin. at the Sha’erqin Research Base (40°33′N, 111°30′E), located in Tumd Left Banner, Hohhot City, Inner Mongolia, China. This region is characterized as a typical agropastoral ecotone, with an annual mean temperature of 6.5 °C and an annual mean precipitation of approximately 350 mm. The predominant soil type in this area is chestnut soil. Using a five-point sampling method, five 10 m × 10 m quadrats were established diagonally across the 5-ha survey plot, and 20 plants were randomly selected from each point, resulting in 100 root samples exhibiting typical root rot symptoms. The field symptoms included plants that failed to green entirely in the spring, with the aboveground parts appearing completely yellow and dead. Additionally, some plants displayed uneven greening, where certain tillers within a single plant greened normally while others remained yellow, with leaves turning yellow and wilting from the tips or margins, resulting in uneven tiller greening and partial clump dieback during spring green-up. Infected plants were carefully uprooted, and adhering soil was gently removed. The samples were placed in sterile A4-sized paper bags, labeled, and transported to the laboratory for isolation within 24 h.

### 2.2 Isolation and purification of pathogens

Fungal isolates were obtained using a standard phytopathological isolation method. Diseased roots were gently rinsed under running tap water to remove adhering soil and then blotted dry with sterile, filter paper. Under aseptic conditions in a laminar flow cabinet, root segments exhibiting typical disease symptoms, including root tips, mid-root sections, and root-crown junctions, were excised and cut into pieces approximately 1–2 cm in length. These pieces were then mixed and surface-sterilized in 75% ethanol for 30 s, followed by three rinses with sterile distilled water. The sterilized root pieces were dried on sterile filter paper and placed on water agar (WA) plates, with 5–8 pieces per plate. The plates were incubated at 25 °C in the dark for 5–7 days. Upon the emergence of mycelia from the margins of the infected root tissues, actively growing hyphal tips were aseptically transferred to potato dextrose agar (PDA) plates for purification. Following 2–3 rounds of single-colony purification, the purified isolates were transferred to PDA slants and stored at 4 °C. Additionally, fungal cultures were preserved at −80 °C ultra-low temperature freezer for long-term storage.

### 2.3 Morphological identification

Each purified isolate was inoculated onto PDA plates and incubated at 25 °C in the dark for 7–14 days. Colony morphology, color, and pigment production were recorded daily. The morphological characteristics of the conidiophores and conidia, including their shape, size, and septation, were examined using an optical microscope (Olympus BX53, Japan). For sporulating isolates 50 conidia were randomly selected from each isolate for measurement, and the mean and standard deviation were calculated. Based on colony morphology and microscopic features, the isolates were preliminarily assigned into taxonomic groups according to Fungi Identification Manual (Wei, 1979) and relevant taxonomic literature.

### 2.4 Molecular identification

#### 2.4.1 Genomic DNA extraction

Representative isolates from each morphological group were selected for genomic DNA extraction using a modified cetyltrimethylammonium bromide (CTAB) method. The isolates were cultured on potato dextrose agar (PDA) plates and incubated at 25 °C for 5–7 days. Approximately 0.1 g of mycelium was harvested, transferred to a sterile mortar, and ground into a fine powder in liquid nitrogen. The powdered mycelium was transferred to a 2 mL microcentrifuge tube, containing 700 μL of preheated CTAB extraction buffer (2% CTAB, 100 mmol/L Tris-HCl [pH 8.0], 20 mmol/L EDTA, and 1.4 mol/L NaCl) and incubated at 65 °C for 1 h with gentle inversion every 15 min. An equal volume of chloroform: isoamyl alcohol (24:1, v/v) was then added, mixed thoroughly, and centrifuged at 12,000 rpm for 10 min. The supernatant was transferred to a new microcentrifuge tube, mixed with an equal volume of isopropanol and incubated at −20 °C for 30 min to precipitate the DNA. After centrifugation at 12,000 rpm for 10 min, the supernatant was discarded, and the DNA pellet was washed twice with 70% ethanol, air-dried, and dissolved in 50 μL of TE buffer (10 mmol/L Tris-HCl, 1 mmol/L EDTA, pH 8.0). DNA quality was assessed by 1% agarose gel electrophoresis, and DNA concentration was determined using a NanoDrop 2000 spectrophotometer (Thermo Fisher Scientific, USA). The DNA samples were stored at −20 °C until further use.

#### 2.4.2 PCR amplification and sequencing

Polymerase chain reaction (PCR) was performed using genomic DNA as the template to amplify the internal transcribed spacer (ITS) region of the ribosomal DNA, the translation elongation factor 1 α gene (*TEF1α*), and the second largest subunit of RNA polymerase II (*RPB2*), using universal primer pairs as listed in **Table 1**. The PCR reaction mixture, with a total volume of 25 μL, comprised 12.5 μL of 2× Taq PCR Master Mix (Tiangen, Beijing), 1.0 μL each of the forward and reverse primers (10 μmol/L), 1.0 μL of genomic DNA template (approximately 50 ng), and sterile double-distilled water to the final volume. PCR amplification consisted of denaturation at 94 °C for 5 min, followed by 35 cycles of denaturation at 94 °C for 30 s at the temperature specified for each primer pair (**Table 1**) annealing for 30 s, and extension at 72 °C for 1 min, with a final extension at 72 °C for 10 min. PCR products were separated by electrophoresis on 1.5% agarose gels and subsequently submitted to Sangon Biotech (Shanghai, China). for bidirectional sequencing.

**Table 1:** Primers used in this study.

Locus	Primer name	Primer sequence (5′→3′)	Annealing temperature (°C)	Reference
ITS	ITS1	TCCGTAGGTGAACCTGCGG	55	(White et al., 1990)
ITS	ITS4	TCCTCCGCTTATTGATATGC	55	(White et al., 1990)
TEF-1α	EF1-728F	CATCGAGAAGTTCGAGAAGG	58	(Carbone and Kohn, 1999)
TEF-1α	EF1-986R	TACTTGAAGGAACCCTTACC	58	(Carbone and Kohn, 1999)
RPB2	RPB2-5F	GAYGAYMGWGATCAYTTYGG	55	(Liu et al., 1999)
RPB2	RPB2-7cR	CCCATRGCTTGYTTRCCCAT	55	(Liu et al., 1999)

#### 2.4.3 Sequence analysis and species identification

Forward and reverse DNA sequences were manually edited and assembled to generate consensus sequences. The assembled sequences were compared with those available in the National Center for Biotechnology Information (NCBI) nucleotide database using the BLASTn to identify closely related taxa. Reference sequences of closely related fungal species were selected based on sequence similarity, query coverage, and taxonomic reliability. For phylogenetic analysis, ITS sequences of ex-type and reference isolates were retrieved directly from NCBI GenBank. For TEF1α and RPB2, reference sequences were obtained from publicly available whole-genome assemblies of type strains using TBtools (Feng et al., 2026), based on the corresponding gene models. Together these sequences constituted the reference dataset used for phylogenetic analyses (**Table 2**). All consensus and reference sequences, were imported into the MEGA version 11 software. Multiple sequence alignments were performed using the Clustal W algorithm with the default parameters. Phylogenetic trees were constructed using the maximum-likelihood (ML) method with 1,000 bootstrap replicates to assess node support. At least three closely related species were included as reference taxa for each genus, and *Lasiodiplodia theobromae* was used as the outgroup (Kumar et al., 2016). Phylogenetic trees were visualized by Chiplot online software (https://www.chiplot.online/). Species identification was based on an integrated analysis of colony morphology, micromorphological characteristics, sequence similarity and phylogenetic relationships.

**Table 2:** Isolates included in this study and their GenBank accession numbers.

Taxon	ITS (isolate)	ITS-GenBank ID:	GENOME (isolate)	GenBank ID:
*Curvularia spicifera*	M3	OR237623.1	FR9030	GCA_026262435.1
*Microdochium bolleyi*	F-85	OM670107.1	J235TASD1	GCA_001566295.1
*Fusarium equiseti*	D1	PX454369.1	S.F-5	GCA_052857265.1
*Mortierella alpina*	LiJJF2	MK014152.1	CGMCC 20262	GCA_029449245.1
*Phaeosphaeria nodorum*	CBS 457.81	KY090647.1	SN15	GCA_000146915.2
*Fusarium solani*	Cc_50	KM017134.1	SB1	GCA_023522795.1
*Fusarium redolens*	Fus 9	KP726904.1	MPI-CAGE-AT-0023	GCA_020744475.1
*Fusarium oxysporum*	MUOC-19	OP199094.1	Fo47	GCA_013085055.1
*Lasiodiplodia theobromae*	HA1102	ON787769.1	AM2As	GCA_012971845.1
*Fusarium acuminatum*	G5	PQ580219.1	1A	GCA_038181435.1
*Fusarium nygamai*	SS-CU4	PZ118607.1	MRC8546	GCA_001262555.1
*Curvularia geniculata*	TJED45	PZ794750.1	P1	GCA_016162275.1
*Phaeosphaeria avenae*	CBS 290.69	MH859307.1	18332-5	GCA_022559985.1
*Microdochium nivale*	200566	KT736220.1	CDWWTD02	GCA_977970205.2
*Microdochium paspali*	HK-ML-1371	KJ569513.1	I16	GCA_031142775.1
*Mortierella polycephala*	CBS 327.72	MH860490.1	KOD948	GCA_016098165.1
*Mortierella antarctica*	Moran01	PZ682956.1	KOD1229	GCA_015849325.1
*Curvularia lunata*	CBS 730.96	NR_138223.1	W3	GCA_005212705.1

### 2.5 Pathogenicity assay

#### 2.5.1 Inoculum preparation

Each test isolate was inoculated onto PDA plates and incubated at 25 °C in the dark for five days. Mycelial plugs (5 mm in diameter) were excised from the actively growing margins of the colonies using a sterile cork borer. An oat grain inoculum was prepared according to previously described methods for pathogenicity assays of chickpea root rot (Geraminasab et al., 2023; Jia et al., 2025). Briefly, oat grains were rinsed with tap water to remove dust, soaked in sterile water for 24 h, and drained to remove excess moisture. Approximately 100 g (wet weight) of soaked oat grains was placed into each 500 mL Erlenmeyer flask and sterilized by autoclaving at 121 °C for 30 min on two consecutive occasions separated by 24 h. After cooling, each 100 g batch of oat grains was inoculated with 10 mycelial plugs (5 mm diameter) and incubated in the dark at 25 °C for 14 days. The flasks were shaken every two days to promote uniform fungal colonization of the oat grains.

#### 2.5.2 Inoculation and seedling preparation

A soil-mix inoculation method was used. Prior to mixing with soil, a subsample of the colonized oat grains was rinsed with sterile water, and the spore concentration of the resulting suspension was determined according to the method described by (Wang et al., 2024) and (Wang et al., 2023). Only oat grains with a spore load of approximately 1 × 10^7^ conidia mL^-1^ were used for inoculation, ensuring that the initial fungal biomass was consistent across all isolates. The qualified colonized oat grains were then thoroughly mixed with sterilized soil at a ratio of 5:1 (v/v, sterilized soil: colonized oat grains). The conidial suspension was used solely for quantification of the spore load and was not applied as inoculum.

*Stipa bungeana* seeds were surface-sterilized using a 1% sodium hypochlorite solution for 5 min, followed by five rinses with sterile water. The disinfected seeds were incubated at 25 °C in the dark for 48 h to facilitate germination. Seeds exhibiting uniform radicle emergence were selected and sown directly into pots (10 cm in diameter, 12 cm in height) containing the prepared infested soil mixture at a density of 10 seeds per pot. Each fungal isolate was tested using, three replicate pots (30 seedlings per isolate), and the entire experiment was repeated independently three. Following sowing, all pots were maintained in a greenhouse at 25 °C under a 12 h light/12 h dark photoperiod with, 60–70% relative humidity. The pots were watered regularly to maintain adequate soil moisture.

#### 2.5.3 Disease assessment

Seed germination and seedling development were monitored daily. Disease assessments were performed at 45 days post-inoculation (dpi). Disease incidence(percentage of diseased plants), mortality,(percentage of dead plants), and overall plant growth were recorded. After the first assessment, the aboveground portions of the plants were clipped to a height of approximately 5 cm using sterile scissors. The plants were then returned to the greenhouse under the same environmental conditions. A second assessment was conducted at 80 dpi, during which disease incidence, plant mortality, and plant growth were evaluated using the same criteria.

#### 2.5.4 Pathogen re-isolation

At both 45-dpi and 80-dpi, fungi were re-isolated from the roots of symptomatic plants following the methods described in Sections 2.2 and 2.3. The recovered isolates were further confirmed by their morphological characteristics and by sequencing the internal transcribed spacer (ITS) region to satisfy Koch’s postulates.

### 2.6 Statistical analysis

Disease incidence and mortality data were analyzed using SPSS version 26.0. The experiment was conducted independently three times, with 30 seedlings per isolate in each replicate. Data from all three replicates were pooled for analysis, individual seedlings were considered the experimental unit. Disease incidence and mortality were analyzed using chi-square tests, with Fisher’s exact tests used when expected frequencies were below 5. All percentage data were calculated directly from the count data (number of diseased or dead seedlings/total number of seedlings) and were not transformed before analysis. Differences were considered statistically significant at α = 0.05.

## 3 Results

### 3.1 Field symptoms of root rot on *Stipa bungeana*

**Figure 1:**
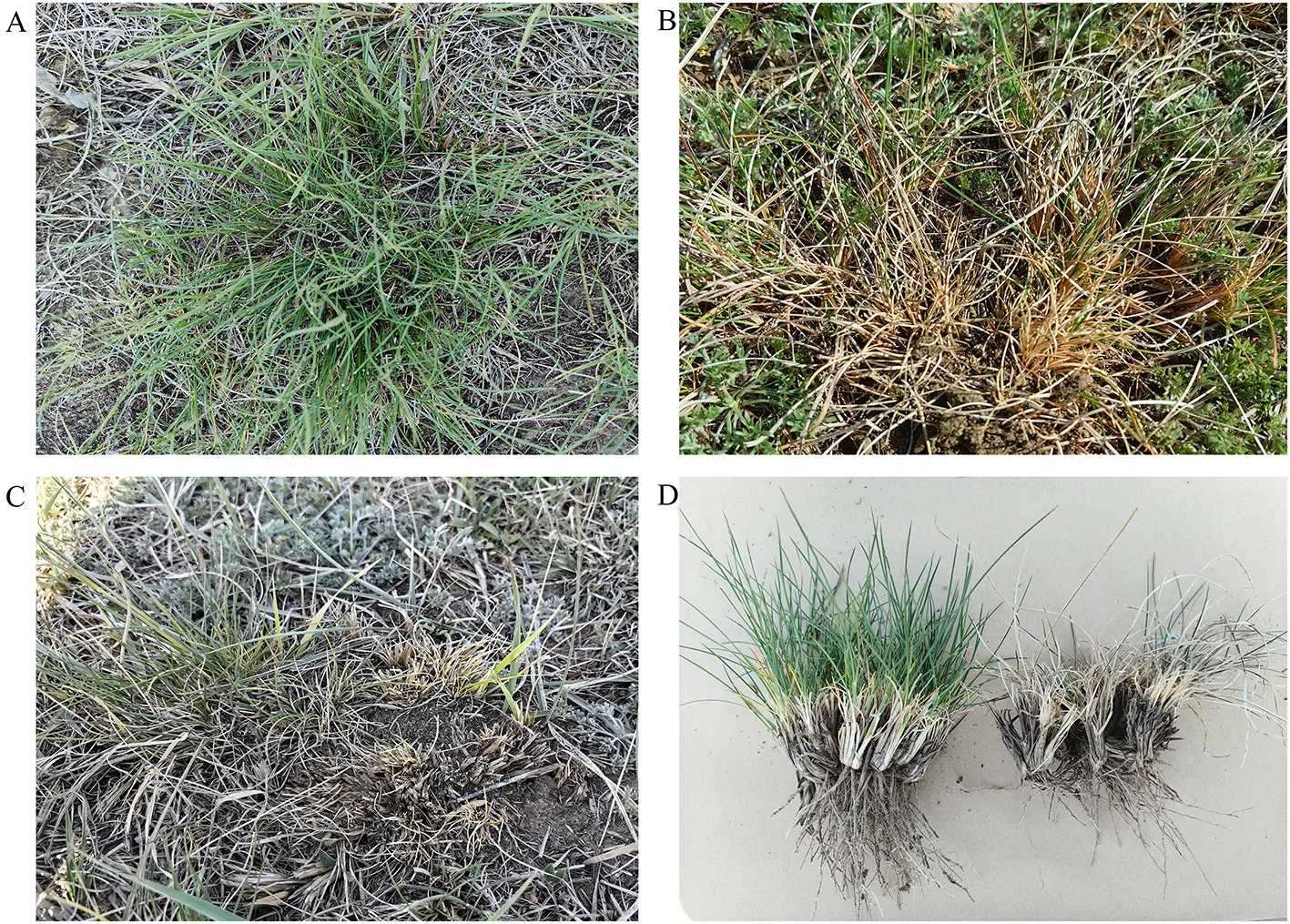
Field symptoms of root rot in Stipa bungeana. **(A)** Healthy plants grown under field conditions; **(B)** chlorosis, yellowing, and wilting of the aboveground parts of diseased plants; **(C)** abnormal tillering, chlorosis, and subsequent death of aboveground tissues in diseased plants; and **(D)** root comparison between a healthy plant (left) and a diseased plant (right). Diseased plants exhibit reduced root biomass, browning, and epidermal rot.

In early May 2025, typical root rot symptoms were observed in *S. bungeana* plants at the Sha’erqin Research Base. **Figure 1A** illustrates a healthy *S. bungeana* plant exhibiting normal growth under field conditions. In contrast, **Figure 1B** depicts a diseased plant with severe chlorosis and death of the aboveground parts during spring. **Figure 1C** illustrates uneven regreening in diseased plants, in which some tillers developed normally whereas others remained yellow. In addition, leaf yellowing and withering began at the tips or margins, showing uneven greening and partial tiller mortality within individual clumps. **Figure 1D** compares the root systems of healthy and diseased plants. Diseased plants exhibited extensive root browning, cortical decay and a marked reduction in root number, whereas healthy plants possessed intact, white root systems. Root rot symptoms were ubiquitous throughout the experimental steppe plot, with diseased clumps distributed uniformly rather than sporadically. The widespread necrosis of root systems resulted in stunted regrowth, tiller death and reduced vegetation cover, potentially increasing the risk of degradation in local agropastoral grasslands.

### 3.2 Morphological identification of fungal isolates

**Figure 2:**
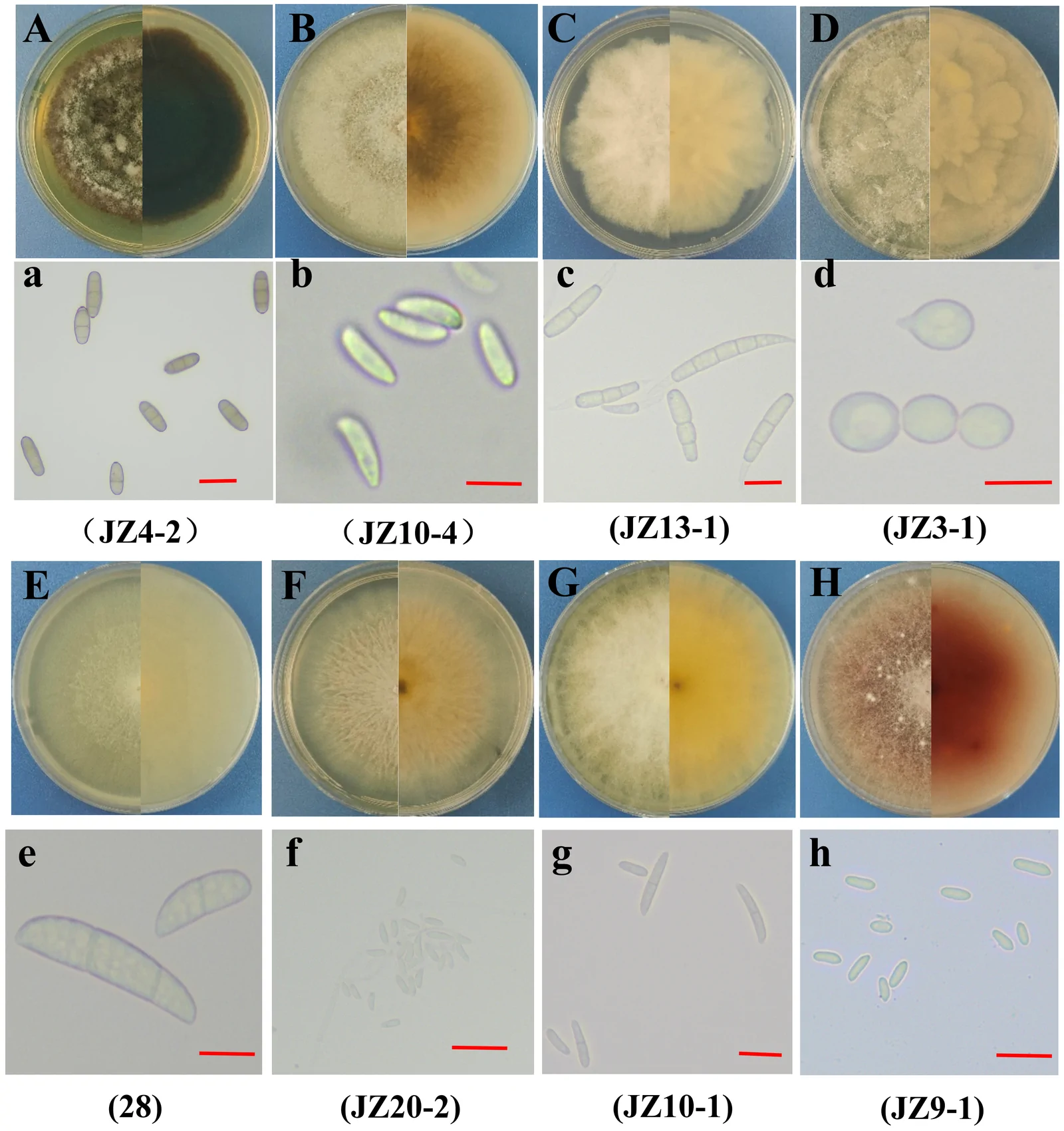
Morphological characteristics of the representative isolates. Upper panels (uppercase letters) show the colony morphology of the isolates grown on PDA medium, with each plate displaying the obverse (left half) and reverse (right half) of the same culture; lower panels (lowercase letters) display conidial morphology. The isolate IDs are presented below each panel. Scale bar = 10 µm.

A total of 76 fungal isolates were recovered from 100 symptomatic root samples. Based on colony morphology and microscopic characteristics, the isolates were preliminarily classified into eight distinct morphological groups. On PDA, representative isolates exhibited colonies of diverse colors, including white, gray, brown, pink, and salmon, with varying colony textures. Microscopic examination revealed distinctive conidiophore and conidial characteristics for each morphological group (**Figure 2**). For example, isolate JZ4–2 produced brown, fusiform conidia with three septa and a curved central cell, whereas, isolate JZ10–4 produced hyaline, one-celled, lunate to ellipsoidal conidia on short conidiophores; and isolates JZ13–1 and 28 produced characteristic macroconidia with foot-shaped basal cells together with microconidia arranged in false heads. Across all eight morphological groups, colony characteristics (pigmentation, texture, and growth rate) and conidial morphology were consistent and diagnostic, allowing reliable preliminary grouping. Full morphological descriptions of each representative isolate are provided in **Table 3**, and representative colony and conidial images are shown in **Figure 2**.

**Table 3:** Tentative morphological descriptions of fungal isolates purified from *S. bungeana* roots infected with root rot disease.

Code	Colony morphology				Microscopic morphology	
	Form	Elevation	Texture	Pigmentation	Microconidia	Macroconidia/Conidia
JZ4-2	Irregular	Raised	Cottony	Raised areas with white mycelium, which later produce black-green to black-yellow pigmentation	Cylindrical, with rounded ends, transparent, and aseptate.	Oblong oval or cylindrical, light brown to brown in color, with 2–3 septa.
JZ10-4	Circular	Raised	Velvety	Light brown to honey-colored, with white edges	No Microconidia	Lunate or sickle-shaped, hyaline, one-celled, with thin walls.
JZ13-1	Irregular	Raised	Velvety	White mycelium	hyaline, ovoid, fusiform (ellipsoidal), or slightly curved	Slightly curved
JZ3-1	Irregular	Flat	Velvety	White mycelium	No Microconidia	Circular or spherical, sometimes slightly curved or irregular in shape, hyaline, one-celled.
JZ20-2	Filamentous	Raised	Velvety	White mycelium	No Microconidia	Cylindrical to fusiform, constricted at both ends, hyaline, usually with 1–3 septa.
28	Circular	Raised	Velvety	White mycelium	Ellipsoidal to ovoid	Spindle-shaped to lanceolate
JZ10-1	Filamentous	Raised	Velvety	Light yellow	Ovoid or cylindrical, typically with 0 to 1 septum.	Sickle-shaped or elongated cylindrical, with the upper cell broader and the apical cell hook-like, bearing 3 to 5 septa.
JZ9-1	Circular	Raised	Velvety	Red and white colors with a fluffy mycelium, and the edges are white	Oval, elliptical, or reniform, colorless, unicellular.	Sickle-shaped, slightly curved with tapering ends, hyaline, multicellular, and typically having 3 to 5 septa.

### 3.3 Molecular identification and phylogenetic analysis

**Figure 3:**
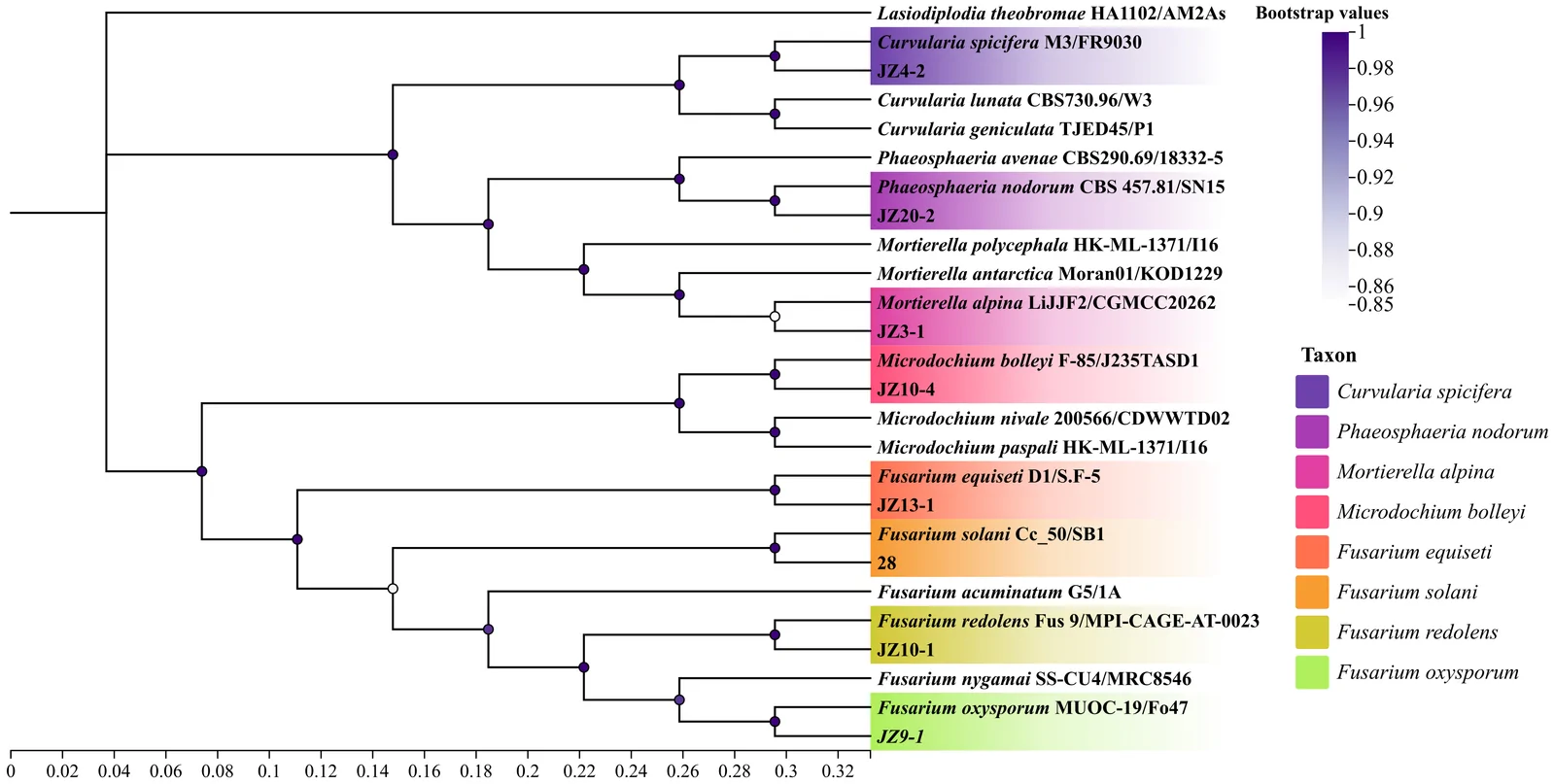
Maximum-likelihood phylogenetic tree based on combined ITS, TEF1α, and RPB2 sequences, showing relationships among the 8 representative isolates and reference taxa. Numbers at nodes represent ML bootstrap values (1,000 replicates).*Lasiodiplodia theobromae* was used as the outgroup.

Representative isolates of each morphological type were sequenced for the internal transcribed spacer (ITS) region of ribosomal DNA, translation elongation factor 1-alpha (TEF1α), and the second largest subunit of RNA polymerase II (RPB2) genes. Using the expanded reference dataset (**Table 2**), a multi-locus phylogenetic tree was constructed using the maximum-likelihood method based on the combined ITS, TEF1α, and RPB2 sequences (**Figure 3**). The tree clearly resolved the eight isolates into well-supported monophyletic clades corresponding to *Curvularia spicifera*, *Microdochium bolleyi*, *Fusarium equiseti*, *Mortierella alpina*, *Phaeosphaeria nodorum*, *Fusarium solani*, *Fusarium redolens*, and *Fusarium oxysporum*. In the phylogenetic tree rooted with *L. theobromae*, all eight isolates formed well-supported monophyletic clades with their reference isolates, confirming their placement within the corresponding genera and species. The inclusion of at least three reference species per genus improved phylogenetic resolution, and all major clades received bootstrap support values of ≥ 70%.

### 3.4 Composition and abundance of isolated taxa

**Figure 4:**
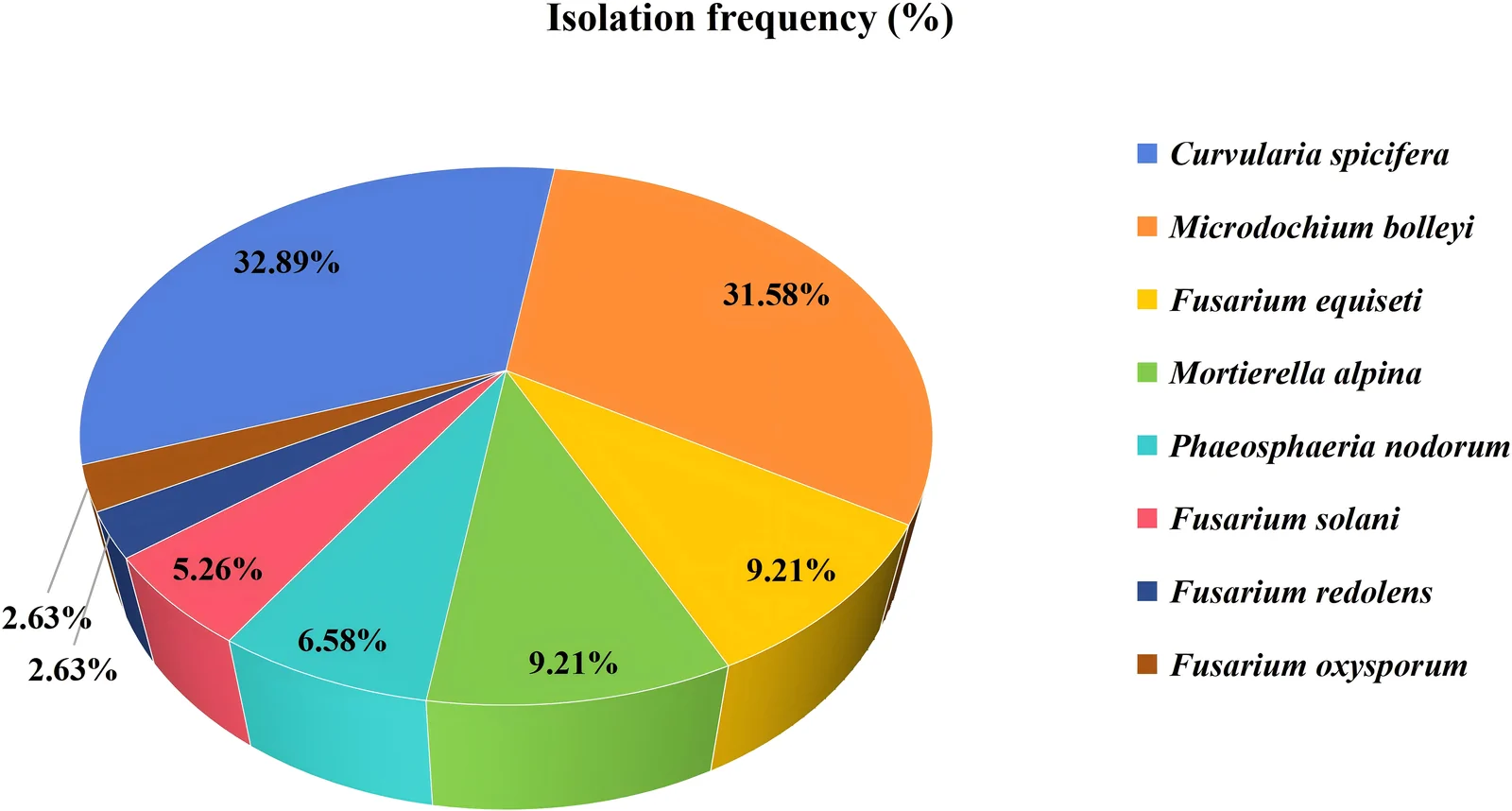
Proportion of each fungal taxon among the 76 isolates from *S. bungeana* root rot samples.

Among the 76 fungal isolates, *Curvularia spicifera* and *Microdochium bolleyi* were the predominant species, accounting for 25 (32.89%) and 24 (31.58%) isolates, respectively. The remaining six taxa collectively represented 35.52% of the total isolates. Within the genus *Fusarium*, 15 isolates (19.73%) were identified, comprising *F. equiseti* (7 isolates), *F. solani* (4 isolates), *F. redolens* (2 isolates), and *F. oxysporum* (2 isolates). The remaining taxa included *Phaeosphaeria nodorum* (5 isolates) and *Mortierella alpina* (7 isolates) (**Figure 4**; detailed isolate counts are represented in **Supplementary Table 1**).

### 3.5 Pathogenicity tests

**Figure 5:**
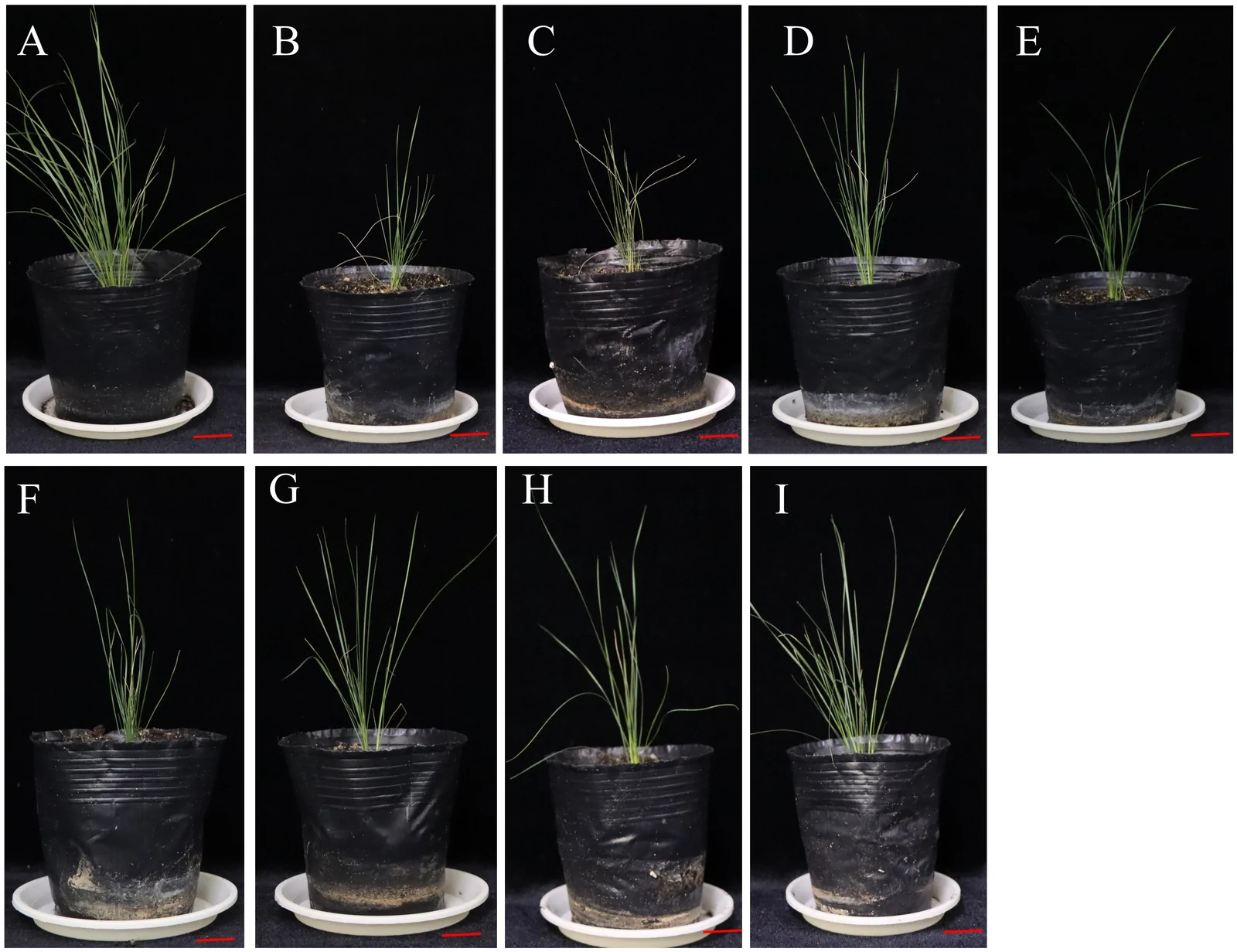
Symptom phenotypes of *Stipa bungeana* seedlings inoculated with different fungal isolates at 45 days post-inoculation (dpi). **(A)** Non-inoculated healthy control; **(B)** *Curvularia spicifera*-inoculated seedling; **(C)** *Microdochium bolleyi*-inoculated seedling; **(D)** *Fusarium equiseti*-inoculated seedling; **(E)** *Mortierella alpina*-inoculated seedling; **(F)** *Phaeosphaeria nodorum*-inoculated seedling; **(G)** *Fusarium solani*-inoculated seedling; **(H)** *Fusarium redolens*-inoculated seedling; and **(I)** *Fusarium oxysporum*-inoculated seedling. Severe seedling wilting and growth stunting were induced by *C. spicifera* and *M. bolleyi*, respectively. Moderate to mild disease symptoms were observed in seedlings inoculated with *Fusarium* species, whereas only slight or no obvious symptoms were detected in plants infected with the other two fungal taxa. The control seedlings grew vigorously without any disease symptoms during the trial. Scale bar = 5 cm.

**Figure 6:**
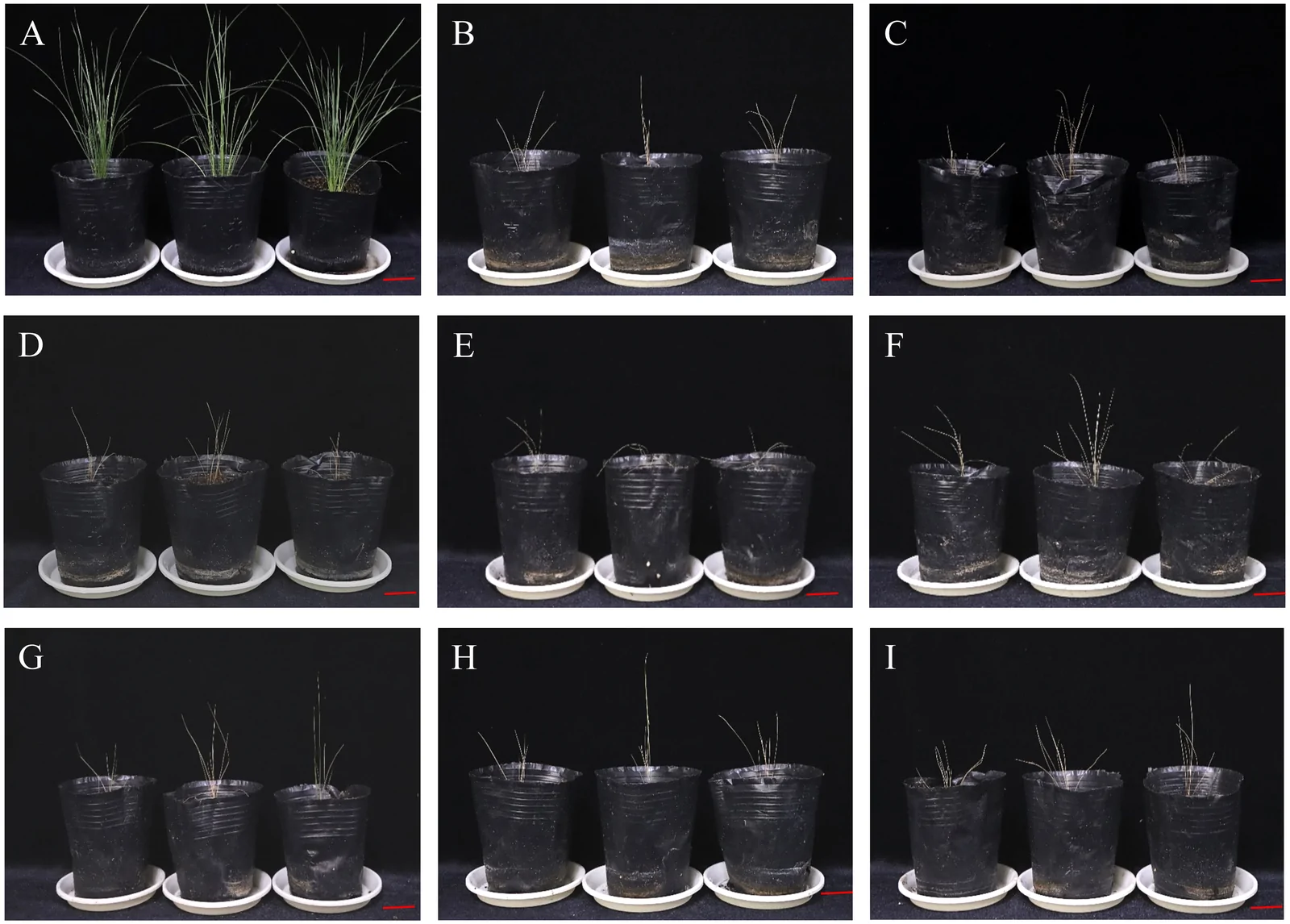
Symptoms on *S. bungeana* seedlings at 80 dpi after inoculation with each of the 8 representative isolates. **(A)** Non inoculated control (healthy); **(B)** *Curvularia spicifera*-inoculated seedling; **(C)** *Microdochium bolleyi*-inoculated seedling; **(D)** *Fusarium equiseti*-inoculated seedling; **(E)** *Mortierella alpina-inoculated* seedling; **(F)** *Phaeosphaeria nodorum*-inoculated seedling; **(G)** *Fusarium solani*-inoculated seedling; **(H)** *Fusarium redolens*-inoculated seedling and **(I)** *Fusarium oxysporum*-inoculated seedling. Scale bar = 5 cm.

The results of pathogenicity tests of eight representative fungal isolates on healthy *S. bungeana* seedlings using the soil mix inoculation method showed that at 45 days post-inoculation (dpi), seedlings inoculated with *C. spicifera* and *M. bolleyi* developed severe browning and cortical rot, accompanied by leaf yellowing, wilting, and reduced plant height compared with non-inoculated control. Seedlings inoculated with the *Fusarium* species and the remaining taxa developed milder symptoms and showed slower disease progression. No statistically significant differences in disease incidence were yet detectable among treatments. Following clipping at 45 dpi, a second disease assessment was conducted at 80 dpi. Seedlings inoculated with *C. spicifera* and *M. bolleyi* exhibited significantly higher disease incidence and mortality than those inoculated with other fungal isolates (**Figures 5**, **6**).

**Figure 7:**
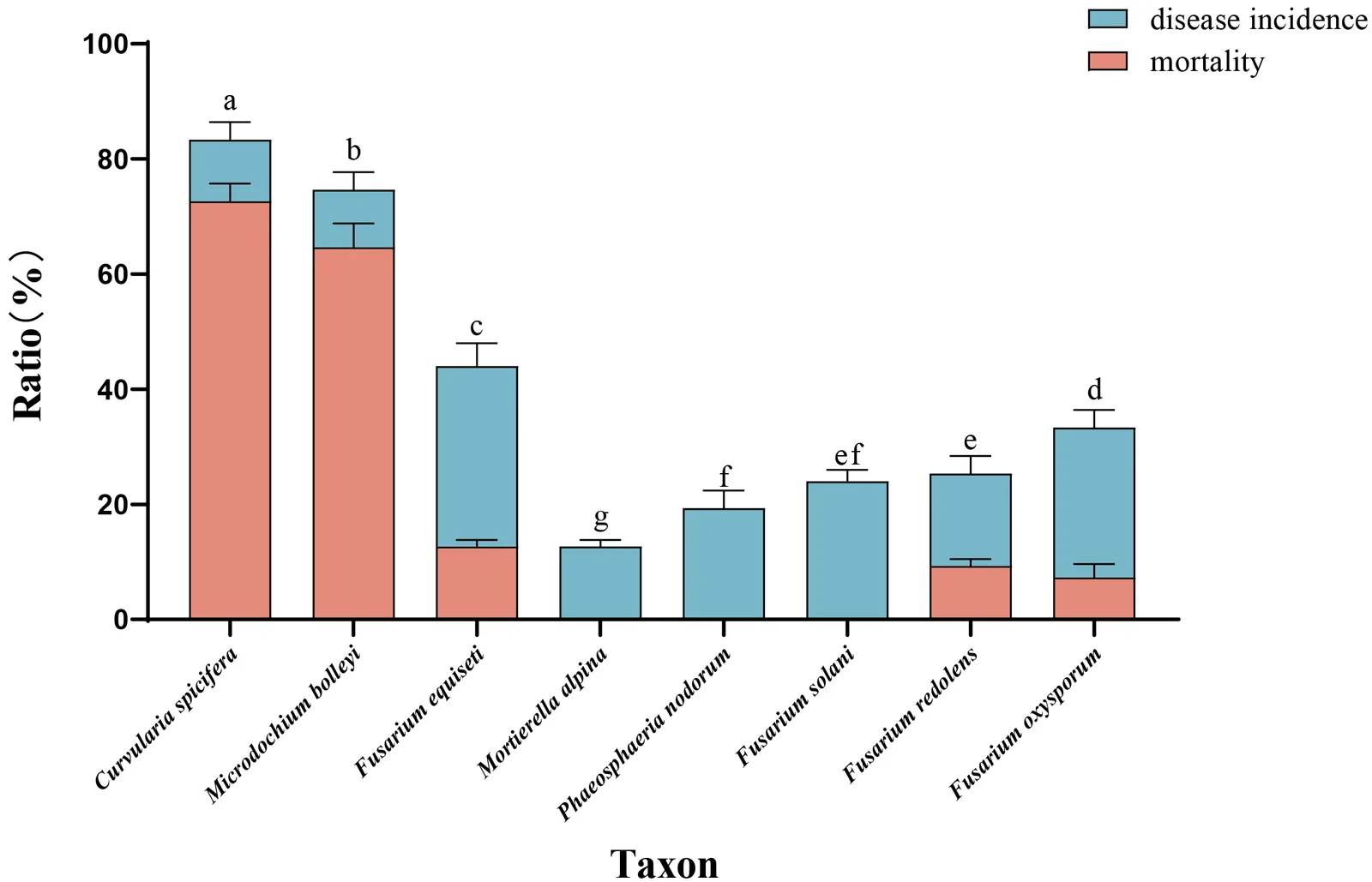
Disease incidence (blue) and mortality (orange) of *S. bungeana* at 80 dpi after inoculation with each isolate. Bars represent the mean ± SD of three independent experiments. Different letters above the bars indicate significant differences (Chi-square test, α = 0.05).

**Figure 7** summarizes the disease incidence and mortality rates for each isolate. *C. spicifera* caused the highest disease incidence (83.33%) and mortality (72.67%) at 80 days post-inoculation (dpi), followed by *M. bolleyi* with an incidence of 74.67% and mortality of 64.67%. The four *Fusarium* species demonstrated moderate disease incidence, ranging from 24.00% to 44.00%, and low mortality between 0% and 12.67%. In contrast, the remaining two taxa induced only weak symptoms, with incidence rates ≤ 20% and no plant mortality. No disease symptoms were observed in the negative control throughout the experiment. Fungi re-isolated from symptomatic roots were identical to the original inoculated isolates, and these were further confirmed by ITS sequence analysis. These results fulfilled Koch’s postulates.

## 4 Discussion

Root rot is a destructive soil-borne disease that directly impairs root function, restricts water and nutrient uptake, and ultimately reduces plant growth and survival (Akber and Fang, 2024; Liu et al., 2025). However, the causal agents of root rot in native grass species in natural steppe ecosystems remain poorly understood. This study provides the first systematic investigation of the pathogens associated with root rot of *S. bungeana*, a dominant grass species in the warm-temperate steppes of northern China. A total of 76 fungal isolates representing eight species were recovered from symptomatic root samples collected from an agro-pastoral ecotone of Inner Mongolia. All eight taxa were confirmed to be pathogenic to *S. bungeana* seedlings under greenhouse conditions, thereby fulfilling Koch’s postulates. Notably, *C. spicifera* and *M. bolleyi*, rather than *Fusarium* species, which are the predominant root rot pathogens of many cultivated forage crops (Zhang et al., 2021; Wang and Li, 2026), were identified as the most abundant and highly virulent pathogens. These findings fill an important knowledge gap in grassland plant pathology and provide a foundation for future disease management in natural steppe ecosystems.

The fungal community associated with root rot of *S. bungeana* differed markedly from those reported for cultivated forage crops in the same region. Previous studies conducted in Inner Mongolia have consistently identified *Fusarium* species, particularly *F. acuminatum*, *F. solani*, *F. oxysporum*, and *F. equiseti* as the principal causal agents of alfalfa root rot (Wang et al., 2023). In the present study, however, *Fusarium* species accounted for only 19.73% of the recovered isolates, and exhibited only moderate to weak pathogenicity. By contrast, *C. spicifera* and *M. bolleyi* together represented 64.47% of all isolates and displayed the greatest virulence. Several factors may explain this difference. First, host specificity drives the formation of distinct pathosystems (Flor, 2025). As a native perennial bunchgrass that has evolved in natural steppe habitats over long periods, *S. bungeana* may have developed unique associations with indigenous fungal pathogens that differ from those affecting intensively cultivated forage legumes. Second, habitat heterogeneity strongly influences soil microbial communities (Baldrian, 2017; Qiao et al., 2025). The sampling site was located in a typical agro-pastoral ecotone with relatively undisturbed steppe vegetation, where the soil microbial community (Sun et al., 2024; Zhang et al., 2024) and the pathogen reservoir differ substantially from those in agricultural systems subjected to long-term cultivation and fertilization (Gao et al., 2023). Such relatively stable natural environments may favor the persistence and dominance of specialized pathogens adapted to the *Stipa* hosts.

*Curvularia* species are cosmopolitan plant pathogens with a broad host range, particularly among the members of the Poaceae family (Cui et al., 2020). Previous reports have documented *C. spicifera* (often formerly known as *Bipolaris spicifera*) causing leaf blight of buffalograss (Amaradasa and Amundsen, 2014) and brown leaf spot of rice (Aslam et al., 2020), and has also been isolated as a seed-borne fungus from wheat (Jeon et al., 2015). In addition, it has been reported as the causal agent of black rot of sweet potatoes (Wu et al., 2025), leaf spot of tomatoes (Ismail et al., 2016), leaf blight and fruit rot of strawberries (Ayoubi et al., 2017). The high virulence of *C. spicifera* observed in *S. bungeana* is consistent with its well-documented pathogenic potential and suggests that it poses a significant threat to the health and stability of steppe ecosystems. An equally important finding was the identification of *M. bolleyi* as a co-dominant and highly virulent pathogen. *Microdochium bolleyi* is commonly associated with cereals and grasses (Stakelienė et al., 2025), where it has been reported to cause root browning and seedling diseases (González and Trevathan, 2000). Although it is often regarded as a weak or opportunistic pathogen in agricultural systems, the present study demonstrates that *M. bolleyi* can function as a highly aggressive pathogen of *S. bungeana*, producing disease severity comparable to that caused by *C. spicifera*. This finding suggests that the pathogenicity of *M. bolleyi* may be strongly influenced by host species or environmental conditions, enabling it to become a major pathogen in natural grassland ecosystems.

The methodological approach adopted in this study also provides a useful framework for future investigations of grassland diseases. The integration of classical morphological observations with multi-locus phylogenetic analyses of ITS, TEF1α, and RPB2 enabled accurate and reliable species identification, particularly for taxonomically challenging genera such as *Curvularia*, in which ITS alone often lacks sufficient discriminatory power (Jeon et al., 2015; Krizsán et al., 2015). Furthermore, the successful completion of Koch’s postulates confirmed the pathogenicity of the recovered fungi. Nevertheless, this study has several limitations. First, all samples were collected from a single sampling site during a single sampling period in May 2025. Although 100 diseased root individuals were collected using five-point sampling to obtain a sufficient number of samples, this intensive single-site sampling strategy cannot fully represent the pathogen community composition across the entire distribution range of *S. bungeana*. Although the Sha’erqin Research Base represents a typical core habitat of this steppe grass and is suitable for systematic baseline investigation of the single spatial and temporal sampling scheme limits the spatial extrapolations and generalizability of conclusion regarding our pathogen composition and virulence. The fungal complex associated with root rot may vary substantially under different climate, soil, and land-use conditions across the agro-pastoral ecotone. In future studies, we will expand sampling to multiple geographically separated sites along the agro-pastoral transition zone and conduct multi-seasonal field surveys to clarify the spatial distribution, seasonal dynamics and regional variation of *C. spicifera* and *M. bolleyi*, the dominant and highly virulent pathogens identified in this study. In addition, pathogenicity tests were conducted under controlled greenhouse conditions, which can not fully reproduce the complex biological and environmental interactions that occur under natural field conditions. Consequently, additional surveys across different regions and long-term field studies are needed to clarify the epidemiology and ecological significance of these pathogens. It should also be noted that disease incidence and mortality were only statistically analyzed at 80 dpi in this study. At 45 dpi, most seedlings showed only mild, incipient symptoms, and virulence differences among isolates had not yet stabilized; clear, statistically distinguishable virulence gradients only developed by 80 dpi after the regrowth period following clipping. Finally, disease assessment in this study was limited to the incidence and mortality. As the first pathogenicity report for this pathosystem, no standardized severity scale exists for *S. bungeana* root rot. Unlike discrete foliar lesions that can be measured individually, root symptoms present as diffuse browning and cortical necrosis across the entire root system, making lesion-length quantification impractical. Therefore, disease incidence and mortality served as the most repeatable metrics for the initial virulence comparison. In future work, we will develop a species-specific severity index based on root rot coverage, root biomass reduction, and aboveground growth inhibition.

In conclusion, this study provides the first comprehensive characterization of the fungal pathogens responsible for root rot of the ecologically important grass species *S. bungeana* in China. The identification of *C. spicifera* and *M. bolleyi* as the dominant and most virulent pathogens substantially advances our understanding of disease dynamics in natural steppe ecosystems. These findings establish a valuable foundation for future studies on pathogen monitoring, ecological impacts, and the development of sustainable disease management strategies for the conservation of *S. bungeana* and the grassland ecosystems it supports.

## Data Availability

The original contributions presented in the study are included in the article/**Supplementary Material**. Further inquiries can be directed to the corresponding authors.

## Supplementary material

Supplementary Table 1 List of fungal taxa isolated from the diseased roots of *Stipa bungeana*.
